# Investing in a healthy lifestyle strategy: is it worth it?

**DOI:** 10.1007/s00038-016-0884-y

**Published:** 2016-09-01

**Authors:** Tarik Benmarhnia, Pierre-Alexandre Dionne, Éric Tchouaket, Alvine K. Fansi, Astrid Brousselle

**Affiliations:** 1 Institute for Health and Social Policy, McGill University, Meredith, Charles House, 1130 Pine Avenue West Montreal, Montreal, QC H3A 1A3 Canada; 2 Centre de recherche de l’hôpital Charles-LeMoyne, Community health sciences department, Université de Sherbrooke, Longueuil, QC Canada; 3 Department de Sciences Infirmières, Universté du Québec en Outaouais, Gatineau, QC Canada; 4 Institut national d’excellence en santé et en services sociaux (INESSS), Quebec, Canada

**Keywords:** Economic evaluation, Public health, Health policy, Burden of disease, Attributable fraction, Return on investment, Risk factors, Economic benefits, Healthy lifestyle habits promotion

## Abstract

**Objectives:**

In Quebec, various actors fund activities aimed at increasing physical activity, improving eating habits and reducing smoking. The objective was to evaluate how effective does the healthy lifestyle habits promotion (HLHP) strategy need to be to make to offset its costs.

**Methods:**

First, we built the logic model of the HLHP strategy. We then assessed the strategy’s total cost as well as the direct health care expenditures associated with lifestyle-related risk factors (smoking, physical inactivity, insufficient intake of fruits and vegetables, obesity and overweight). Finally, we estimated the break-even point beyond which the economic benefits of the HLHP strategy would outweigh its costs.

**Results:**

The HLHP strategy cost for 2010–2011 was estimated at $110 million. Direct healthcare expenditures associated with lifestyle-related risk factors were estimated at $4.161 billion. We estimated that 47 % of these expenditures were attributable to these risk factors.

**Conclusions:**

We concluded that the HLHP strategy cost corresponded to 5.6 % of the annual healthcare expenditures attributable to these risk factors. This study compared the economic value of HLHP activities against healthcare expenditures associated with targeted risk factors.

**Electronic supplementary material:**

The online version of this article (doi:10.1007/s00038-016-0884-y) contains supplementary material, which is available to authorized users.

## Introduction

Both the epidemiological landscape and public health threats have evolved considerably over the past decade in Western countries, moving from risks of infectious disease epidemics to a strong prevalence of lifestyle-related health problems (Brownson et al. 2006). In the United States, for example, health problems related to lifestyle habits (smoking, sedentary lifestyle, poor nutrition, excessive alcohol consumption) account for 900,000 deaths annually, or nearly 40 % of total mortality (Abraham et al. 2009; Cohen et al. 2008). Effective public health programs can increase life expectancy, improve quality of life and reduce health system costs (Goldsmith et al. 2004). Yet despite the available evidence and the recognized health and economic burdens associated with cardiovascular and respiratory diseases, diabetes and cancer, public health programs represent only a very meagre portion of total health spending (Brownson et al. 2006). Neumann et al. (2008) point out that even if the value of public health programs appears obvious in the light of scientific knowledge, the chronic underfunding of public health activities indicates that public is not very aware of their value.

In Quebec, various actors fund activities related to environmental action and education that are aimed at increasing physical activity, improving eating habits and reducing smoking (Ministère de la Santé et des Services sociaux (MSSS) 2008). The objective of our study was to evaluate the economic value of such activities in Quebec which, for purposes of this study, we refer to collectively as the healthy lifestyle habits promotion (HLHP) strategy. As the effectiveness of this large scope policy has not been evaluated, we evaluated how effective does the HLHP strategy need to be to make to offset its costs. More specifically, we compared investments to economic benefits, analysing at what point HLHP costs were outweighed by economic benefits, measured as savings in healthcare expenditures related to a reduction in risk factors targeted by HLHP activities. We specifically define the economic benefits of the HLHP strategy as economic savings in direct healthcare expenditure (i.e. drugs, hospital care, and medical care).

In this article, we first describe our methodology, after which we present the results and discuss the innovative nature and limitations of this approach. This article may be of interest to public health authorities and researchers, both for the information it provides on the economic value of HLHP activities in Quebec as compared to healthcare expenditures associated with targeted risk factors, and for the methodology used to capture information that encompasses a broad range of activities and programs, in a context where actual effectiveness is not known.

## Methods

**Fig. 1:**
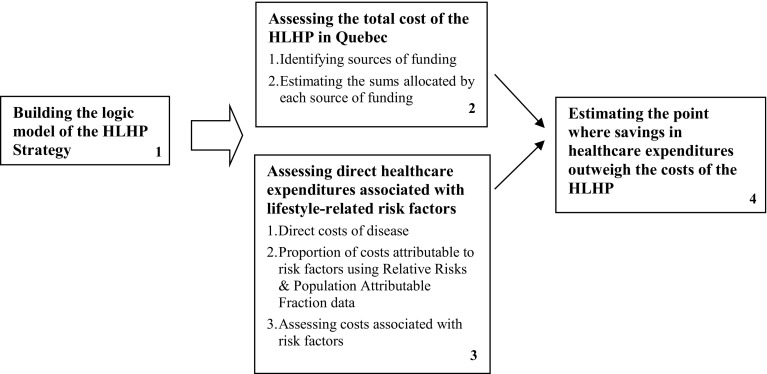
Methodological process. Canada 2016

The methodology (Fig. 1) for this study consisted of: (1) building the logic model for the HLHP strategy and related activities (Brousselle and Champagne 2011); (2) assessing the total cost for the HLHP strategy; (3) assessing direct healthcare expenditures associated with lifestyle-related risk factors (smoking, physical inactivity, insufficient intake of fruits and vegetables, obesity and overweight); and (4) estimating the point at which savings in healthcare expenditures related to unhealthy lifestyle habits outweigh HLHP strategy costs (break-even point). In cases of uncertainty, to obtain a valid and conservative estimate we overestimated HLHP strategy costs and underestimated expenditures attributable to complications related to risk factors.

Our approach has similarities with economic evaluation and program evaluation methods: logic model (Brousselle and Champagne 2011), cost-benefit (Commonwealth Department of Health and Ageing 2003; Drummond et al. 2005), cost-offset type studies (Chiles et al. 1999; Kelly et al. 2005), and cost-consequence analysis (Canadian Agency for Drugs and Technologies in Health (CADTH) 2006; Coast 2004). The methods used in this economic evaluation are, to some extent, similar to those used in the previous studies assessing the economic impact of chronic diseases in other Canadian provinces or other countries (Conference Board of Canada 2010; Katzmarzyk and Janssen 2004; Krueger et al. 2013; Scarborough et al. 2011), and the economic benefits of a water fluoridation program (Tchouaket et al. 2013).

### HLHP strategy logic model development

The logic model provides an exhaustive representation of the resources, activities (programs, interventions), expected effects, and impacts associated with those effects (Drummond et al. 2005; Funnell and Rogers 2011). To build this logic model, we consulted the grey and the scientific literatures. The model underwent two rounds of validation by public health experts and people closely involved in the strategy’s design and coordination.

### Assessing the total cost of Quebec’s HLHP strategy

The strategy we evaluated is made up of many programs and activities funded by various actors at the federal, provincial, regional, and community levels. As such, to identify all the activities aimed at promoting healthy lifestyle habits and quantify how much is allocated to each activity would present a very complex challenge. We were able to get around this problem by identifying primary sources of funding and evaluating the total amounts granted by each of them annually.

To identify the primary funders and the amounts invested, we consulted the grey literature (financial reports of organizations, government reports) and people involved in public health administration. A top down costing approach was selected to estimate the cost of the HLHP strategy. We used the standardized costs of the Programme national de santé publique–PNSP (Quebec Public Health Program), which are primary estimates of the human resources needed at local and regional levels to plan, implement, and coordinate public health activities (Direction générale de la santé publique–DGSP 2010). These data are estimates that are likely higher than what is actually invested in public health in Quebec, making them conservative estimates for determining the break-even point. We used 2010–2011 fiscal year costs for the analyses. The data on the costs of prevention activities were validated by experts working for Quebec’s Ministry of Health and Social Services (MSSS).

### Assessing direct healthcare expenditures associated with lifestyle-related risk factors

#### Cost of diseases associated with unhealthy habits in Quebec

The first step in assessing healthcare expenditures attributable to modifiable risk factors is to calculate the costs of chronic diseases associated with those factors. As illustrated in the logic model, the diagnoses retained for calculating disease costs were those shown in the literature to be associated with the risk factors targeted by the HLHP strategy (smoking, insufficient intake of fruits and vegetables, physical inactivity, obesity and overweight). We did not include in our calculations all the healthcare expenditures attributable to these risk factors, but rather the principal ones, such that the calculated level of effectiveness at which economic benefits surpass HLHP strategy costs is a conservative estimate (higher than what it would be in reality). It should be noted that, in our study, we excluded from the calculation of costs attributable to risk factors: (1) indirect costs (e.g. mortality, lost productivity); (2) costs related to other illnesses associated with the risk factors (e.g. vascular disease related to diabetes, some types of cancers); and (3) other nonmedical outcomes such as improved quality of life and higher self-esteem. Hence, by not quantifying the indirect costs, we considerably underestimated the costs attributable to risk factors.

Using data from the Economic Burden of Illness in Canada (EBIC) studies (Public Health Agency of Canada (PHAC) 2000), we calculated the direct costs of these chronic diseases in Quebec in 2010 for hospitalizations, ambulatory care visits, and medications. This is the same source of data on illness costs that has been used in other studies to determine the costs of some chronic diseases in Canada (Conference Board of Canada 2010; Krueger et al. 2013; Katzmarzyk and Janssen 2004). The data in the EBIC report on the costs of illnesses are the result of complex analyses performed over more than 10 years by the Public Health Agency of Canada (PHAC) on data from several Canadian Institute for Health Information (CIHI) databanks and from self-reported surveys, including the Canadian Community Health Survey (CCHS) (Health Canada 2002). To calculate the costs of illness in 2010, we extrapolated the cost data from 2000 over an 11-year span using CIHI’s National Health Expenditure Database (CIHI 2012). In this way, we obtained a projection of the annual costs of illness in relation to changes in spending in different sectors of health-related activity (e.g. medications, hospitalizations, medical visits).

### Assessing the proportion of the costs attributable to the risk factors

Using cost data attributable to specific effects (medications, hospitalizations, medical visits), we calculated the number of cases of illness attributable to the risk factors under consideration (smoking, insufficient intake of fruits and vegetables, physical inactivity). For this, we calculated the proportions attributable to each risk factor for all the illnesses.

Many epidemiologic studies have assessed the link between lifestyle habits or changes (such as smoking cessation) and chronic disease incidence. In this study, we considered various exposures and health effects. Based on the relationships expressed between a given exposure and a corresponding effect—for example, with relative risks (RR)—and using the prevalence of this exposure, it is possible to calculate a population attributable fraction (PAF) using the formula 1.

1 $${\text{PAF}}\,\, = \,\, Pe\,\,({\text{RR}} - 1)/Pe\,\,({\text{RR}} - 1)\,\, + \,\,1$$

where *Pe* represents the exposure prevalence (in a given context) and RR the relative risk, expressing the relationship between exposure and effect.

PAFs were calculated separately for each exposure/health effect relationship (RR) considered in this study. From each PAF it was then possible to calculate a number of cases attributable to one exposure or, in this situation, the proportion of disease costs attributable to the risk factor. We calculated adjusted PAFs following the approach described in studies by Benichou (2001), Hanley (2001), and Steenland and Armstrong (2006) to consider the level of exposure for a given risk factor (e.g. smokers, occasional smokers, former smokers, and never smokers) and multi-exposure for one given health effect (e.g. the combined impact of smoking and physical inactivity and obesity). PAF estimates were adjusted for level of exposure when data was available (e.g. smoker, occasional smoker, former smoker, never smoker). By doing this dual adjustment, we avoided double-counting the economic burden of the risk factors (Krueger et al. 2013). To calculate the number of cases (for the health effects considered) and the proportion of costs attributable to each exposure, we undertook a multi-stage process of systematic review and data analysis, details of which are presented in the supplemental material.

### Evaluating the point at which savings in healthcare expenditures outweigh the costs of the HLHP strategy

We then estimated the point at which savings in healthcare expenditures related to unhealthy lifestyle habits outweighed HLHP strategy costs. To estimate this break-even point (%), we used formula 2:

2 $$\frac{{{\text{Total amount allocated to HLHP strategy}}\,\, (\$ )}}{{{\text{Direct health expenditures attributable to targeted risk factors}}\,\, (\$ )}}$$

The break-even threshold represents the minimal level of savings required to offset the cost of the HLHP strategy; in fact, the real threshold is most certainly below the calculated threshold, since we did not include in our calculations all the costs attributable to risk factors.

#### Discounting

To take into account the fact that effects occur several years after investments in prevention activities, we modelled the occurrence of effects on different time horizons (0, 5, 10, 15 and 20 years) by discounting the costs attributable to the risk factors using discount rates recommended by the Center for Public Health Excellence at National Institute for Health and Clinical Excellence (NICE 2011) (1.5 % for health benefits and 3.5 % for costs) and using Canadian Institute for Health Information (Canadian Institute for Health Information (CIHI) 2012) data on health expenditures in Canada to project the increases in healthcare expenditures attributable to these illnesses. Between 2000 and 2010, health expenditures (in 1997 constant dollars) increased by 4.4 % annually. We assumed costs attributable to the risk factors would increase at the same rate (4.4 %) as health expenditures in Canada over recent years. Different sensitivity analyses were conducted that are presented in the supplemental material.

## Results

### The logic model

**Fig. 2:**
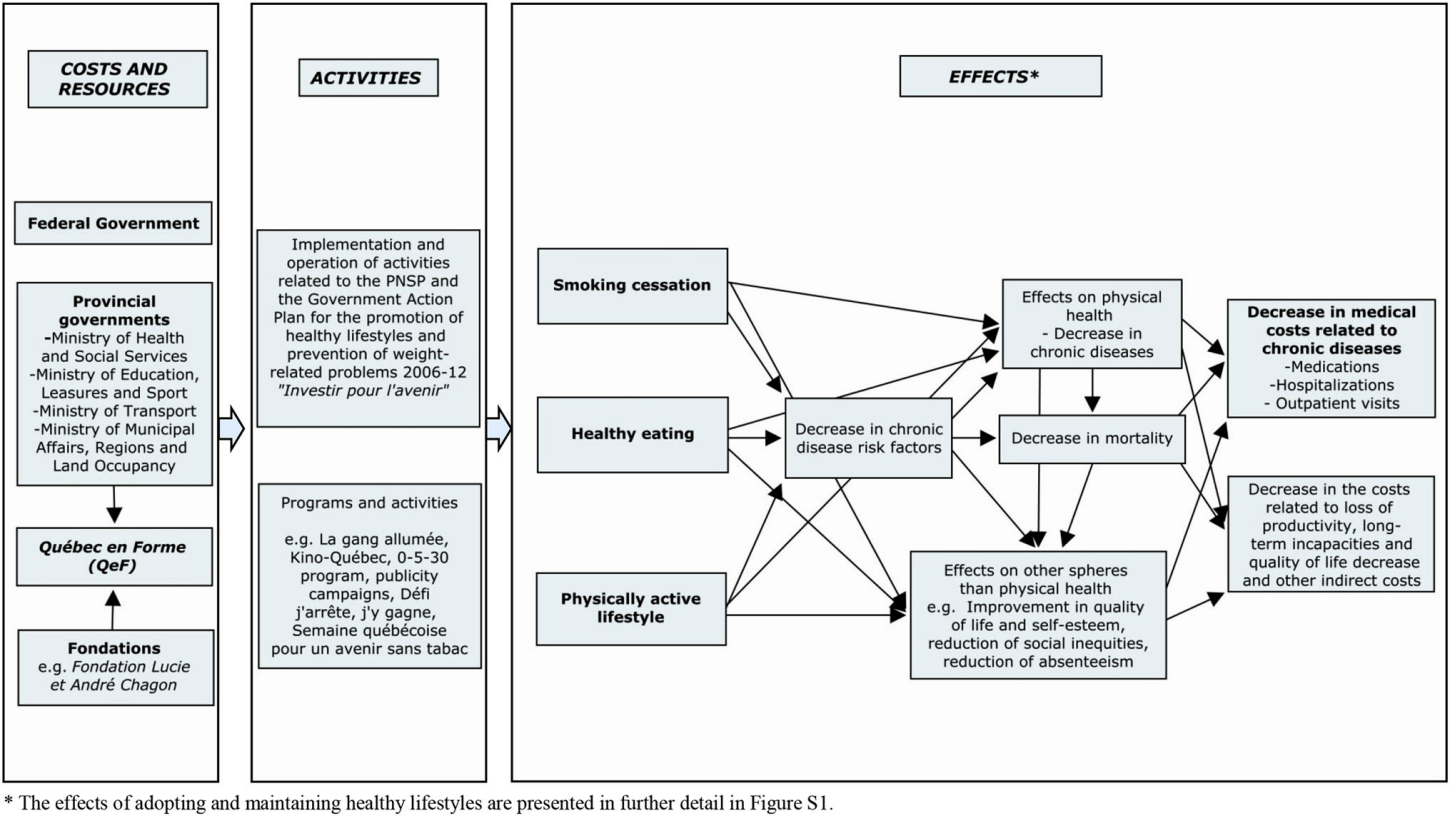
Logic model of the healthy lifestyle habits promotion strategy in Quebec. Canada 2016

Given the scope and complexity of the HLHP strategy, the logic model was divided into two different models: one presenting the resources, activities, and key effects (Fig. 2); and another depicting the chain of effects of adopting healthy lifestyle habits (Figure S1). It is important to recall that these models were built with the idea that only health expenditures avoided due to reduction in chronic illnesses (direct costs of risk factors) would be estimated for the economic analysis.

### Cost of the HLHP strategy

Healthy lifestyle habits promotion and chronic disease prevention activities were grouped into five categories based on type of funding. For each category, Table S2 presents all the subcategories of activities, their funding sources, examples of interventions, and their costs for the year 2010–2011.

Total cost was estimated at approximately $110 million for the 2010–2011 fiscal year (amounts given in this paper are in Canadian dollars). As presented in Table S2, some costs could not be quantified for some sources of funding. These costs seem to be negligible compared to the main sources of funding. Furthermore, certain costs may be overestimated; in particular the PNSP’s standardized costs. For the sensitivity analyses, we assumed the costs of the HLHP strategy could vary between $90 million and $150 million.

### Direct healthcare expenditures associated with lifestyle-related risk factors

#### Cost of diseases associated with unhealthy habits in Quebec

The total direct healthcare expenditures of chronic diseases selected for our study in Quebec in 2010 are presented in Table 1. Using the health expenditure data from CIHI and data from the EBIC survey, we estimated the total all-cause direct healthcare expenditures to be $21.72 billion in 2010 in Quebec—and more specifically, as related to the diseases targeted in our study, $379 million for diabetes mellitus, $3.45 billion for cardiovascular diseases, over $700 million for asthma and chronic obstructive pulmonary diseases (COPD), and over $360 million for lung, colorectal, and breast cancers. Based on our forecasts, the chronic diseases selected in our economic model (subcategories presented in Table 1) were responsible for $4.161 billion of direct healthcare spending in 2010. Therefore, chronic diseases related to modifiable risk factors were responsible for an important part (approximately one fifth) of total direct healthcare expenditures in 2010 in Quebec.

**Table 1:** Economic burden of illness by diagnostic category in Quebec

Code	Cause	Direct costs*^, †^			
		Drugs	Hospital care	Medical care	All direct costs
W000	All causes	$6,267,268,479	$10,801,577,118	$4,647,884,743	$21,716,730,340
W060	A. Malignant tumours				
W064	4. Colon and rectal cancer	$3,682,912	$112,028,852	$16,223,691	$131,935,455
W067	7. Tracheal, bronchial and lung cancer	$4,750,870	$123,715,726	$23,135,123	$151,601,720
W069	9. Breast cancer	$13,768,738	$42,144,588	$21,509,603	$77,422,929
W079	C. Type 2 diabetes	$147,840,004	$132,547,663	$99,296,278	$379,683,944
W104	G. Cardiovascular diseases				
W106	2. Hypertensive disease	$741,822,143	$57,843,016	$169,159,096	$968,824,255
W107	3. Ischemic heart disease	$290,308,382	$505,568,277	$167,814,583	$963,691,242
W108	4. Cerebrovascular disease	$22,588,070	$396,509,597	$38,292,634	$457,390,301
W111	H. Respiratory conditions				
W112	1. Chronic obstructive pulmonary disease	$83,672,505	$229,684,077	$81,379,783	$394,736,365
W113	2. Asthma	$206,389,146	$69,830,400	$59,673,740	$335,893,285
W125	L. Musculoskeletal diseases				
W127	2. Osteoarthritis (arthrosis)	$57,525,894	$138,020,356	$43,047,497	$238,593,747

Canada 2016

* Forecasts based on health expenditure data from Canadian Institute for Health Information (CIHI) and Economic Burden of Illness in Canada (EBIC) data from the Public Health Agency of Canada (Katzmarzyk and Janssen 2004; Kelly et al. 2005; Krueger et al. 2013).

^†^The chronic diseases selected in our economic model (subcategories A4, A7, A9, C, G2, G3, G4, H1, H2, L2) were responsible for $4.161 billion of direct healthcare spending in 2010

### Assessing the proportion of the costs attributable to the risk factors

The studies included in our literature review and the selected RRs for each risk factor are presented in Table S1. Relative risks, the prevalence of exposure, and the PAFs of total chronic disease costs for each risk factor and each chronic disease are presented in Table 2. According to the CCHS survey, the prevalence of smoking among persons aged 12 years and older in Quebec was 18.9 % among males and 15.7 % among females in 2009–2010. In that same year, 56.7 % of men and 39.7 % of women had insufficient intake of fruits and vegetables (fewer than five portions daily), and 58.2 % of men and 63.5 % of women did not reach the recommended level of physical activity. The prevalence of obesity and overweight were 17.5 and 41.0 %, respectively, in men and 15.4 and 27.1 % in women (Table 2).

**Table 2:** Assessment of Population attributable fractions (PAF) of total direct healthcare costs

Chronic diseases	Risk factors (RF)	Men					Women				
		RR^a^	(95 % CI^b^)		*P* ^c^ (%)	Adj. PAF^d^ (%)	RR^a^	(95 % CI^b^)		*P* ^c^ (%)	Adj. PAF^d^ (%)
(1) Pulmonary diseases											
Chronic obstructive pulmonary disease	Smoker	4.11	3.28	5.15	18.9	59.9	3.28	2.35	4.58	15.7	39.4
	Occasional smoker	2.14	1.87	2.46	6.3		2.14	1.87	2.46	4.9	
	Former smoker	2.97	2.63	3.34	42.3		1.61	1.46	1.77	38.8	
	Insufficient consumption of fruits and vegetables	1.32	1.09	1.67	56.7	15.2	1.32	1.09	1.67	39.7	11.1
Asthma	Smoker	1.70	1.30	2.20	18.9	13.6	1.30	0.80	2.30	15.7	6.3
	Occasional smoker	1.40	1.20	1.60	6.3		1.40	1.20	1.60	4.9	
	Obesity	1.43	1.14	1.79	17.5	13.6	1.78	1.36	2.32	15.4	15.8
	Overweight	1.20	1.08	1.33	41.0		1.25	1.05	1.49	27.1	
(2) Cancers											
Lung cancer	Smoker	8.96	6.63	12.1	18.9	73.0	7.58	5.36	10.7	15.7	68.1
	Former smoker	3.85	2.77	5.34	42.3		3.85	2.77	5.34	38.8	
	Insufficient consumption of fruits and vegetables	1.21	1.04	1.45	56.7	10.6	1.21	1.04	1.45	39.7	7.7
Breast cancer	Physical inactivity						1.49	1.00	2.27	63.5	23.7
	Insufficient consumption of fruits and vegetables						1.12	1.01	1.25	39.7	4.5
Post-menopausal breast cancer	Obesity						1.15	1.08	1.23	15.4	4.3
	Overweight						1.08	1.03	1.23	27.1	
Colon and rectum cancer	Obesity	1.95	1.59	2.39	17.5	27.3	1.66	1.52	1.81	15.4	18.3
	Overweight	1.51	1.37	1.67	41.0		1.45	1.30	1.62	27.1	
	Physical inactivity	1.26	1.10	1.47	58.2	13.1	1.40	1.13	1.74	63.5	20.3
(3) Cardiovascular diseases											
Stroke	Obesity	1.26	1.07	1.48	17.5	6.2	1.26	1.07	1.48	15.4	5.1
	Overweight	1.05	0.93	1.17	41.0		1.05	0.93	1.17	27.1	
	Insufficient consumption of fruits and vegetables	1.20	1.12	1.30	56.7	10.4	1.05	0.96	1.14	39.7	2.0
	Physical inactivity	1.25	1.15	1.35	58.2	12.7	1.22	1.14	1.32	63.5	12.3
	Smoker	1.43	1.35	1.52	25.2	15.3	1.72	1.59	1.86	20.6	17.6
	Former smoker	1.17	1.05	1.88	42.3		1.17	1.05	1.88	38.8	
Ischemic heart diseases	Obesity	1.72	1.51	2.24	17.5	19.7	3.10	2.81	3.43	15.4	35.1
	Overweight	1.29	1.18	1.41	41.0		1.80	1.64	1.98	27.1	
	Insufficient consumption of fruits and vegetables	1.11	1.02	1.22	56.7	5.9	1.32	0.95	1.82	39.7	11.1
	Physical inactivity	1.10	0.96	1.30	58.2	5.5	1.25	1.09	1.45	63.5	13.7
	Smoker	1.60	1.26	2.02	25.2	12.8	3.22	2.47	4.22	20.6	34.0
	Former smoker	0.99	0.69	1.42	42.3		1.15	0.92	1.44	38.8	
Hypertension	Obesity	1.84	1.51	2.24	17.5	20.7	2.42	1.59	3.67	15.4	28.3
	Overweight	1.28	1.10	1.50	41.0		1.65	1.24	2.19	27.1	
	Physical inactivity	1.47	1.11	2.70	58.2	21.5	1.47	1.11	2.70	63.5	23.0
	Smoker	1.15	1.03	1.27	25.2	6.7	1.15	1.03	1.27	20.6	5.8
	Former smoker	1.08	1.01	1.15	42.3		1.08	1.01	1.15	38.8	
(4) Metabolic diseases											
Type II diabetes	Obesity	6.48	5.17	8.13	17.5	61.9	8.38	5.46	12.85	15.4	65.1
	Overweight	2.63	2.09	3.32	41.0		3.69	2.52	5.40	27.1	
	Insufficient consumption of fruits and vegetables	1.20	1.11	1.32	58.2	10.4	1.20	1.11	1.32	63.5	11.3
	Physical inactivity	1.04	0.85	1.27	56.7	2.2	1.04	0.85	1.27	39.7	1.6
(5) Musculoskeletal diseases											
Osteoarthritis	Obesity	4.20	2.76	6.41	17.5	56.2	1.96	1.88	2.04	15.4	26.7
	Overweight	2.76	2.05	3.70	41.0		1.80	1.75	1.85	27.1	

Canada 2016

^a^Relative risk

^b^Confidence interval

^c^Prevalence

^d^Population attributable fraction

Table 2 presents PAF estimates adjusted for multiple exposures to each disease (combined effect of having multiple risk factors for the same disease); the crude estimates of costs associated with each risk factor, and the adjusted costs associated with these risk factors for specific diseases assessed using the adjusted PAF estimates (Table 3). The targeted risk factors were responsible for over $1 billion of cardiovascular disease costs (respectively, $522, $436, and $161 million for ischemic heart diseases, hypertension, and stroke) in Quebec in 2010. In addition, they were also responsible for most of the direct healthcare expenditures for COPD ($222 million), lung cancer ($111 million), and diabetes mellitus ($259 million). Looking specifically at each risk factor, smoking, obesity and overweight, insufficient intake of fruits and vegetables, and physical inactivity were responsible for $699 million, $951 million, $403 million, and $232 million in direct healthcare expenditures, respectively, in 2012.

**Table 3:** Direct healthcare costs associated to lifestyle-related risk factors

Chronic diseases	Direct healthcare costs (CAN $2010)	Risk factors (RF)	Adj. PAF^a^ (%)	Costs attributable to risk factors ($)	Total costs adjusted for multiple exposition ($)
(1) Pulmonary diseases					
Chronic Obstructive Pulmonary disease	$394,736,365	Smoking	49.52	$195,464,225	$221,658,019
		Insufficient consumption of fruits and vegetables	13.14	$51,887,048	
Asthma	$335,893,285	Smoking	9.88	$33,195,061	$77,751,443
		Obesity and overweight	14.72	$49,442,607	
(2) Cancers					
Lung cancers	$151,601,720	Smoking	70.56	$106,973,175	$111,056,160
		Insufficient consumption of fruits and vegetables	9.15	$13,869,767	
Breast cancers	$77,422,929	Physical inactivity	4.55	$3,520,702	$21,058,496
		Insufficient consumption of fruits and vegetables	23.73	$18,373,294	
Post-menopausal breast cancers	$61,164,114	Obesity and overweight	4.29	$2,621,537	$2,621,537
Colon and rectum cancers	$131,935,455	Obesity and overweight	22.71	$29,963,004	$47,049,393
		Physical inactivity	16.76	$22,106,957	
(3) Cardiovascular diseases					
Stroke	$457,390,301	Smoking	16.48	$75,356,930	$161,321,080
		Insufficient consumption of fruits and vegetables	6.17	$28,230,351	
		Physical inactivity	12.48	$57,064,937	
		Obesity and overweight	5.63	$25,751,457	
Ischemic heart diseases	$963,691,242	Smoking	23.55	$226,961,558	$522,497,040
		Insufficient consumption of fruits and vegetables	8.57	$82,558,945	
		Physical inactivity	9.67	$93,144,537	
		Obesity and overweight	27.50	$264,972,860	
Hypertension	$968,824,255	Smoking	6.25	$60,584,638	$436,253,706
		Insufficient consumption of fruits and vegetables	22.24	$215,504,459	
		Obesity and overweight	24.59	$238,211,364	
(4) Metabolic disease					
Type II diabetes	$379,683,944	Insufficient consumption of fruits and vegetables	1.89	$7,161,712	$258,622,744
		Physical inactivity	10.85	$41,212,151	
		Obesity and overweight	63.55	$241,271,621	
(5) Musculoskeletal diseases					
Osteoarthritis	$238,593,747	Obesity and overweight	41.21	$98,326,933	$98,326,933
Direct costs of chronic diseases	$4,160,937,358			Total costs attributable to risk factors	$1,958,216,550

Canada 2016

^a^Population attributable fraction

After adjusting for multiple exposures, the sum of all direct healthcare expenditures attributable to the selected risk factors was estimated at $1.958 billion in 2010, corresponding to nearly half the total expenditures for related chronic diseases ($4.16 billion). In fact, these risk factors were responsible for 47 % of all direct healthcare expenditures associated with the chronic diseases included in our study.

### The point at which healthcare expenditure savings outweigh HLHP strategy costs

Using the baseline estimate of the costs of the HLHP strategy ($110 million), we estimated that the costs of HLHP activities in Quebec represented 5.62 % of the total healthcare expenditures attributable to the risk factors ($1.958 billion). This estimate does not include these diseases’ indirect costs, such as the economic impact of mortality and lost productivity due to long-term and short-term disabilities, nor the direct costs of other diseases associated with unhealthy lifestyle choices. Hence, the real break-even point is likely to be lower than the estimate we calculated.

### Discounting the effects

Table S3 presents the results of the analyses of the impact of discounting the effects (3.5 % discount rate) and accounting for increases in healthcare spending over different time horizons. If the economic benefits (savings from disease avoidance) are assumed to occur 10, 15, or 20 years after the HLHP activities, the break-even point decreases to 5.15, 4.93, and 4.72 %, respectively.

## Discussion

Quebec’s HLHP strategy is a large-scale program encompassing all activities related to environmental actions and education that are aimed at increasing physical activity, improving eating habits, and reducing tobacco use (Ministère de la Santé et des Services sociaux (MSSS 2008). The costs of these activities were estimated at $110 million in 2010. Very few studies have comprehensively estimated the total cost of health promotion activities. One other study that attempted to quantify the costs of such activities in Quebec produced a higher estimate of $127 million in 2008 ($31 million for smoking cessation programs and $96.3 million for obesity prevention activities) (Manuel et al. 2009). In addition, the objective of this paper was to assess how effective does Quebec’s HLHP strategy need to be to make to offset its costs. This analysis goes beyond a separated calculation of the two sides of the costs. By estimating the point at which healthcare expenditure savings outweigh HLHP strategy costs (5.62 %), we were thus able to highlight the potential amplitude of further public health investments.

In this study, healthcare expenditures attributable to the risk factors targeted by the HLHP strategy were estimated at $1.958 billion in 2010. As such, the cost of the HLHP strategy ($110 million) represents only 5.6 % of the healthcare expenditures associated with these risk factors. In another study conducted in Manitoba, Krueger et al. (2013) estimated the direct costs attributable to the same risk factors as considered in our study to be $490 million in 2008, substantially lower than our estimate. However, when adjusted for the number of inhabitants per province, the costs associated with these risk factors in the Manitoba study ($409 per capita; total population of 1,197,774 in Manitoba in 2008) are higher than our estimates ($247 per capita; total population of 7,923,365 in Quebec) (Statistics Canada 2014). This difference may be explained by a different study design and different risk factors retained for the assessment of total healthcare expenditures. In the study by Krueger et al. (2013), the indirect costs of these risk factors, notably the costs of lost productivity, long-term disability, and mortality—which were not included in our study—represented 70 % of total costs ($1.114 billion out of a total $1.6 billion). This highlights the magnitude of the indirect costs associated with these risk factors in Canada. In our study, if the indirect costs associated with these risk factors represented 70 % of the total costs, the total costs would be $6.53 billion.

### Challenges

Several challenges emerged during this evaluation (Drummond et al. 2008; Shiell et al. 2008; Weatherly et al. 2009). First, a health promotion strategy is not a single, confined intervention; it is in fact a number of actions, interventions, and programs with a common orientation, each with its own costs and funding, and leading to widespread and long-term effects with complex causalities (Craig et al. 2008). Developing a complete and accurate description of all activities related to the HLHP strategy as well as their sources of funding represented a challenge in itself. Nevertheless, the fact that the HLHP strategy included all activities related to healthy habits, allowed us to work with attributable risks for estimating the potential healthcare cost savings at the provincial level.

Second, the element of time is likely to influence these results (Soler et al. 2016), although it is difficult to foresee in what way. First, illness-related costs will be avoided over a certain number of years, but it is impossible to estimate this time horizon with any precision. We performed sensitivity analyses assuming avoided expenditures at time horizons of 5, 10, 15, and 20 years by discounting, with various discount rates, attributable costs and adjusting for expenditure increases over time. The results of these analyses indicated that taking into account the time horizon has a limited impact, as the discount rates are similar to the annual rates of increases in health expenditures in Canada (4.4 %). Such analyses also run into certain methodological limitations, such as the difficulty of forecasting time horizons for effects and of anticipating changes in disease management approaches. These methodological limitations may influence, in either direction, the costs of treatment and future savings generated by the HLHP strategy. Moreover, identifying activity costs is a challenge when dealing with a strategy encompassing many activities. Some costs may not have been listed and others may have been overestimated or underestimated. To counteract this limitation, we used high estimates of activity costs in modelling to be sure of obtaining conservative results. We also performed sensitivity analyses to assess the impacts of program and activity cost variations on our results.

### Strengths

To estimate the portion attributable to each risk factor, we used measures of association drawn from a literature review (See Table S1 in Supplemental material), with strict selection criteria that would most closely approximate the characteristics of the population of Quebec. This approach meant that the selected measures of association could not be directly attributed to the study population. Nevertheless, to limit this bias and to estimate the proportions that could be attributable, we developed an approach that allowed for systematic selection of measures of association and incorporated a sensitivity analysis. This approach can be replicated in other contexts and makes it possible, using criteria from the studies identified in the literature review, to prioritize RRs in ways that will ensure the study population is represented as accurately as possible.

The originality of our study is that it compares investments in health promotion activities against amounts devoted to treating major diseases associated with the targeted risk factors. We have no effectiveness data, but we believe that achieving the healthy lifestyle objectives set by the MSSS would result in considerable savings that would completely finance the HLHP strategy. Although it is difficult to change people’s lifestyle habits, efforts over recent years to reduce smoking in Quebec have lowered smoking prevalence from 27 % in 2003 to 24 % in 2009–10 (DGSP 2012). In comparison with the burden that lifestyle habits related diseases will represent in coming years according to current epidemiological trends, investments in HLHP activities seem relatively small (Poirier and Jobin 2011). Our results indicate as it is likely that even a small effectiveness in risk reduction could produce important savings for the healthcare system in terms of costs averted.

### Conclusion

Lifestyle-related illnesses have become a major public health concern around the world over recent years, and the growing prevalence all around the world is a serious concern for public health authorities (Geneau et al. 2010). In this article, we show that it is possible to conduct an economic evaluation of a large-scale health promotion strategy encompassing multiple interventions, activities, and programs. The methodology we used is situated at the intersection of several fields. We combined methods from the fields of evaluation, economic evaluation, and epidemiology. We were able to assess the economic value of the HPHL strategy by comparing its costs to the healthcare expenditures associated with diseases related to targeted risk factors.

Our study demonstrates that the financial risk of investing in health promotion activities aimed at improving lifestyle habits is small when compared with the financial burden of diseases associated with the targeted risk factors.

## Electronic supplementary material

Below is the link to the electronic supplementary material.

Supplementary material 1 (DOCX 377 kb)
