# Supplementary material for: Investing in a healthy lifestyle strategy: is it worth it?
Source: Int J Public Health. 2016 Sep 1;62(1):3–13. doi: 10.1007/s00038-016-0884-y (PMC5288452; doi:10.1007/s00038-016-0884-y)
Supplement: Supplementary file 1 — Supplementary material 1 (DOCX 377 kb) [file 38_2016_884_MOESM1_ESM.docx]

**Supplemental Material – International Journal of Public Health**

**Investing in a Healthy Lifestyle Strategy: Is it worth it?**

**Authors:** Tarik Benmarhnia ^1*^, Pierre-Alexandre Dionne^2*^, Éric Tchouaket^3^, Alvine K. Fansi^4^, Astrid Brousselle^2^

(1) Institute for Health and Social Policy, McGill University. QC, Canada

(2) Charles-LeMoyne Hospital Research Centre, University of Sherbrooke. QC, Canada

(3) Department of Nursing, University of Quebec in Outaouais. QC, Canada

(4) Institut national d'excellence en santé et en services sociaux (INESSS)

* these 2 authors equally contributed to this work

Email addresses: [tarik.benmarhnia@mcgill.ca](mailto:tarik.benmarhnia@mcgill.ca); [pierre-alexandre.Dionne@usherbrooke.ca](mailto:pierre-alexandre.Dionne@usherbrooke.ca); [Eric.Tchouaket@uqo.ca](mailto:Eric.Tchouaket@uqo.ca); [afansi@yahoo.com](mailto:afansi@yahoo.com); [astrid.brousselle@usherbrooke.ca](mailto:astrid.brousselle@usherbrooke.ca)

**Corresponding author**: Tarik Benmarhnia

Mailing address: Institute for Health and Social Policy, McGill University. Meredith, Charles, House, 1130 Pine Avenue West Montreal, Quebec H3A 1A3, Canada. Tel: +15146384393, E-mail: [tarik.benmarhnia@mcgill.ca](mailto:tarik.benmarhnia@mcgill.ca)

**Appendix 1: Scoping review of systematic reviews, meta-analyses, and cohort studies for the assessment of the proportion of the costs attributable to each risk factors**

**Description of the process used for the systematic reviews.**

1. We selected the most appropriate relationship between the exposure targeted by the HLHP (Healthy Lifestyle Habits Promotion) strategy and the selected health effects. We then conducted a scoping review of systematic reviews, meta-analyses, and cohort studies using the following selection criteria:
   - RRs (Relative Risks) from systematic reviews and meta-analyses;
   - RRs for North American or European populations (Western populations), to consider populations comparable to that of Quebec;
   - RRs from studies published after 2000 inclusively;
   - RRs expressed for the same exposure measurements; e.g. as the HLHP policy was aimed at increasing the prevalence of physical activity, measured as 30 minutes of activity per day, we retained studies expressing physical activity as 30 minutes of activity per day;
   - RRs stratified by sex.
2. We organized the RRs collected through the review into a hierarchy according to these standards. When it was not possible to meet all the criteria, we proceeded as follows:
   - If no data were available in systematic reviews or meta-analyses, we used RRs from North American cohorts.
   - If no data were available after 2000, we retained the last systematic reviews or meta-analyses before 2000.
   - If no data were available stratified by sex, we used the same RRs for men and women.
3. We prioritized the articles according to the criteria described above and chose one RR for each relationship (stratified by sex when possible) as most appropriate to represent the population of Quebec targeted by the identified actions of the HLHP strategy and kept the other RRs for the sensitivity analyses. Studies selected in our literature review of RR are presented in Table 1 (Appendix 1).
4. We calculated adjusted PAFs (Population Attributable Fraction) using the RRs selected based on our literature review and the exposure prevalence data from the 2009–2010 CCHS (Canadian Community Health Survey) survey.^28^ These PAFs were multiplied by the costs of disease to obtain the costs attributable to each risk factor.

**Appendix 2: Objectives of the healthy lifestyle habits promotion strategy in Quebec, Canada**

**A – Healthy lifestyle**

- 5% increase in the proportion of people eating 5 or more proportions of fruits and vegetables daily.
- 5% increase in the proportion of people reaching the recommended level of physical activity
- Reduce the rate of smoking to 13% in young people and to 16% in people aged 15 and over
- Reduce the prevalence of food insecurity to less than 4.5%

**B – Chronic diseases and risk factors**

- Limit the increase of diabetes to 30%
- 2% decrease in the prevalence of obesity and 5% decrease in the prevalence of overweight
- Reduce to 3%/year mortality and morbidity rates due to Cardiovascular Diseases (CVD)
- 25% reduction in mortality rate due do breast cancer
- 15% reduction in mortality and morbidity rates due to asthma and Chronic Obstructive Pulmonary Disease (COPD).

**Sources:**

- Ministère de la Santé et des Services sociaux (MSSS), 2008. Programme national de santé publique 2003-2012, mise à jour 2008 [National Program of Public Health 2003-2012, update 2008]. Gouvernement du Québec, Québec. Available at : <http://publications.msss.gouv.qc.ca/acrobat/f/documentation/2008/08-216-01.pdf>
- Alliance for the Prevention of Chronic Disease, 2002. Building the case for the prevention of chronic disease. Disease Intervention Division, Centre for Chronic Disease Prevention and Control, Health Canada, Ottawa.
- Ministère de la Santé et des Services sociaux (MSSS), 2006. Investir pour l'avenir - Plan d'action gouvernemental de promotion des saines habitudes de vie et de prévention des problèmes reliés au poids 2006-2012 [Government action plan for the promotion of healthy lifestyles and prevention of weight-related problems, Investing for the Future, 2006-2012]. Gouvernement du Québec, Québec. Available at : <http://www.saineshabitudesdevie.gouv.qc.ca/extranet/pag/index.php?le-pag>
- National Public Health Partnership (NPHP), 2001. Preventing Chronic Disease: A Strategic Framework [online]. Background paper, Melbourne, Victoria (Australia). Available at: [www.nphp.gov.au](http://www.nphp.gov.au)

**Figure S1. Causal chain effects of adopting and maintaining healthy lifestyles on the occurrence of chronic diseases and related costs. Canada 2016**


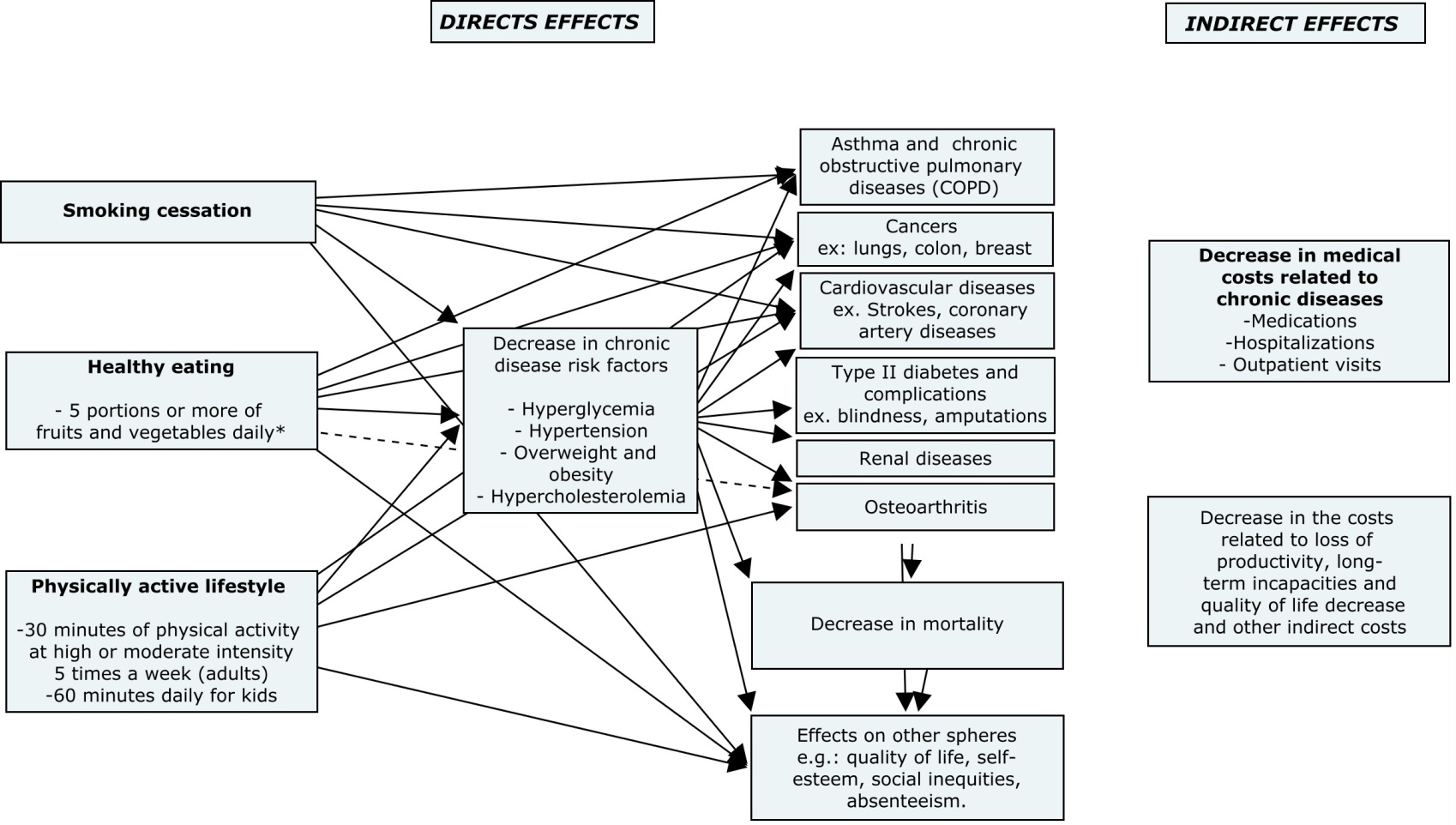


**= Possible association; = Established association**

**Reduction of**

**chronic diseases**

***The consumption of fruit and vegetables is a good indicator of the quality of eating in a population**

**References:**

- Alliance for the Prevention of Chronic Disease, 2002. Building the case for the prevention of chronic disease. Disease Intervention Division, Centre for Chronic Disease Prevention and Control, Health Canada, Ottawa.
- National Public Health Partnership (NPHP), 2001. Preventing Chronic Disease: A Strategic Framework [online]. Background paper, Melbourne, Victoria (Australia). Available at: [www.nphp.gov.au](http://www.nphp.gov.au)
- WHO and FAO (World Health Organization and Food and Agriculture Organization of the United Nations), 2003. Diet, Nutrition, and the Prevention of Chronic Diseases: Report of a Joint WHO/FAO Expert Consultation. Report 916. WHO, Geneva.
- IARC (International Agency for Research on Cancer), 2002. Weight Control and Physical Activity. IARC Press, Lyon, France.

**Table S1. Studies selected in our literature review of relative risks (RR). Canada 2016**

| **Diseases** | | **Main risk factors** | **Studies selected** | **Relative risks (CI 95%)** | |
| --- | --- | --- | --- | --- | --- |
|  |  |  |  | **Men** | **Women** |
| ***1) Pulmonary diseases*** | |  |  |  |  |
|  | **COPD** | Smoker | **Forey et al. 2011 - BMC Pulm Med** | **4.11 (3.28–5.15)** | **3.28 (2.35–4.58)** |
|  |  |  | Chen et al. 2000 - Clin Epidemiol | 3.22 (1.87, 5.53) |  |
|  |  |  | Bednarek et al. 2005 - Respiration | 2.05 (1.13, 3.73) |  |
|  |  |  | Zielinski et al. 2006 - Eur Respir J 2006 | 2.23 (1.99, 2.50) |  |
|  |  |  | Shahab et al. 2006 - Thorax | 2.31 (1.99, 2.68) |  |
|  |  |  | Montnemery et al. 1998 - Respir Med | 2.56 (1.98, 3.31) |  |
|  |  | Occasional smoker | **Shahab et al. 2006 - Thorax** | **2.14 (1.87–2.46)** | **2.14 (1.87–2.46)** |
|  |  |  | Bednarek et al. 2005 - Respiration | 1.58 (0.92, 2.71) |  |
|  |  |  | Sargeant 2000 - Eur Respir J | 1.40 (1.25, 1.58) |  |
|  |  | Former smoker | **Forey et al. 2011 - BMC Pulm Med** | **2.97 (2.63–3.34)** | **1.61 (1.46–1.77)** |
|  |  |  | Bednarek et al. 2005 - Respiration | 1.17 (0.62, 2.23) |  |
|  |  |  | Ho et al. 1999 - J Am Geriatr Soc | 2.31 (1.23, 4.34) |  |
|  |  |  | Forastiere et al. 1998 - Am J Respir Crit Care Med | 2.00 (1.22, 3.27) |  |
|  |  |  | Hozawa et al. 2006 - Chest | 1.87 (1.70, 2,07) |  |
|  |  | Insufficient consumption of fruits and vegetables | **Walda et al. 2002 - Eur J Clin Nutr** | **1.32 (1.09–1.67)** | **1.32 (1.09–1.67)** |
|  |  |  | Varasso et al. 2007 - Thorax | 2.47 (1.11, 5.52) |  |
|  |  |  | Varasso et al. 2007 - Am J Clin Nutr |  | 2.19 (1.19, 4.04) |
|  | **Asthma** | Smoker | **Santillan et al. 2003 - Cancer Causes Control** | **1.7 (1.3–2.2)** | **1.3 (0.8–2.3)** |
|  |  |  | Alanvaja et al. 1992 - Am J Epidemiol | 1.3 (0.8–2.1) | 1.3 (0.8–2.1) |
|  |  | Occasional smoker | **Santillan et al. 2003 - Cancer Causes Control** | **1.4 (1.2–1.6)** | **1.4 (1.2–1.6)** |
|  |  | Obesity | Flaherman et al. 2006 - Arch Dis Child | 1.22 (1.13–1.34) | 1.23 (1.13–1.34) |
|  |  |  | Yuan et al. 2002 - Int J Epidemiol | 1.52 (1.14–2.03) | 1.52 (1.14–2.03) |
|  |  |  | **Guh et al. 2009 - BMC Public Health** | **1.43 (1.14–1.79)** | **1.78 (1.36–2.32)** |
|  |  | Overweight | Flaherman et al. 2006 - Arch Dis Child | 1.45 (0.91–2.31) | 1.45 (0.91–2.31) |
|  |  |  | **Guh et al. 2009 - BMC Public Health** | **1.20 (1.08–1.33)** | **1.25 (1.05–1.49)** |
|  |  |  |  |  |  |
| ***2) Cancers*** | |  |  |  |  |
|  | **Tracheal, bronchial and lung cancers** | Smoker | **Gandini et al. 2008 - Int J Cancer** | **8.96 (6.63, 12.11)** | **7.58 (5.36, 10.73)** |
|  |  |  | Gandini et al. 2008 - Int J Cancer | 10.10 (6.50, 14.6) | 10.10 (6.50, 14.6) |
|  |  | Former smoker | **Gandini et al. 2008 - Int J Cancer** | **3.85 (2.77, 5.34)** | **3.85 (2.77, 5.34)** |
|  |  | Insufficient consumption of fruits and vegetables | **Smith-Warner et al. 2003 - Int J Cancer** | **1.21 (1.04–1.45)** | **1.21 (1.04–1.43)** |
|  | **Breast cancer** | Physical inactivity | Monninkhof et al. 2007 - Epidemiology |  | 1.18 (0.98, 1.41) |
|  |  |  | **Lee et al. 2001 - Cancer Causes Control** |  | **1.49 (1.00, 2.27)** |
|  |  |  | Katzmarzyk et al. 2004 - Can J Appl Physiol |  | 1.31 (1.23, 1.38) |
|  |  |  | Moore et al. 2000 - Epidemiology |  | 1.02(0.78–1.33) |
|  |  | Insufficient consumption of fruits and vegetables | **Aune et al. 2012 - Breast Cancer Res Treat** |  | **1.12 (1.01–1.25)** |
|  | ***Post-menopausal breast cancer*** | Obesity | **Renehan et al. 2008 - Lancet** |  | **1.15 (1.08, 1.23)** |
|  |  |  | Guh et al. 2009 - BMC Public Health |  | 1.13 (1.05–1.22) |
|  |  | Overweight | **Guh et al. 2009 - BMC Public Health** |  | **1.08 (1.03–1.14)** |
|  | **Cancer colorectal** | Obesity | Renehan et al. 2008 - Lancet | 1.35 (1.21,1.50) | 1.13 (1.06, 1.19) |
|  |  |  | **Guh et al. 2009 - BMC Public Health** | **1.95 (1.59–2.39)** | **1.66 (1.52–1.81)** |
|  |  | Overweight | **Guh et al. 2009 - BMC Public Health** | **1.51 (1.37–1.67)** | **1.45 (1.30–1.62)** |
|  |  | Physical inactivity | **Samad et al. 2004 - Colorectal Dis** | **1.26 (1.10–1.47)** | **1.40 (1.13–1.74)** |
|  |  |  | Boyle et al. 2012 - J Natl Cancer Inst | 1.40 (1.20–1.67) |  |
|  |  |  | Katzmarzyk et al. 2004 - Can J Appl Physiol | 1.31 (1.41–1.53) |  |
|  |  |  | Colbert et al. 2001 - Cancer Epidemiol Biomarkers Prev | 2.22 (1.28–3.85) |  |
|  |  |  |  |  |  |
| ***3) Cardiovascular diseases*** | |  |  |  |  |
|  | **Strokes** |  |  |  |  |
|  |  | Obesity | **Strazzulo et al. 2010 - Stroke** | **1.26 (1.07–1.48)** | **1.26 (1.07–1.48)** |
|  |  |  | Guh et al. 2009 - BMC Public Health | 1.51 (1.33–1.72)* | 1.49 (1.27–1.74)* |
|  |  | Overweight | **Strazzulo et al. 2010 - Stroke** | **1.05 (0.93–1.17)** | **1.05 (0.93–1.17)** |
|  |  |  | Guh et al. 2009 - BMC Public Health | 1.23 (1.13–1.34)* | 1.15 (1.00–1.32)* |
|  |  | Insufficient consumption of fruits and vegetables | **He et al. 2006 - Lancet** | **1.20 (1.12–1.30)** | **1.05 (0.96–1.14)** |
|  |  | Physical inactivity | **Li et al. 2012 - Int J Environ Res Public Health** | **1.25 (1.15–1.35)** | **1.22 (1.14–1.32)** |
|  |  |  | Wendel-Vos et al. 2004 - Int J Epidemiol | 1.28 (1.18–1.41) | 1.28 (1.18–1.41) |
|  |  |  | Do lee et al. 2003 - Stroke | 1.25 (1.16–1.35) | 1.25 (1.16–1.35) |
|  |  |  | Diep et al. 2010 - J Women Health | 1.14 (1.06–1.22) | 1.01 (0.93–1.14) |
|  |  |  | Katzmarzyk et al. 2004 - Can J Appl Physiol | 1.60 (1.42–1.80) | 1.60 (1.42–1.80) |
|  |  | Smoker | **Shinton et al. 1989 - BMJ** | **1.43 (1.35-1.52)** | **1.72 (1.59–1.86)** |
|  |  | Occasional smoker | No data |  |  |
|  |  | Former smoker | **Shinton et al. 1989 - BMJ** | **1.17 (1.05–1.88)** | **1.17 (1.05–1.88)** |
|  |  |  |  |  |  |
|  | **Ischemic heart disease** | Obesity | **Guh et al. 2009 - BMC Public Health** | **1.72 (1.51–2.24)*** | **3.10 (2.81–3.43)** |
|  |  | Overweight | **Guh et al. 2009 - BMC Public Health** | **1.29 (1.18–1.41)*** | **1.80 (1.64–1.98)** |
|  |  |  |  |  |  |
|  |  | Insufficient consumption of fruits and vegetables | **HE et al. 2007 - J Human Hypertension** | **1.11 (1.02–1.22)** | **1.32 (0.95–1.82)** |
|  |  |  |  |  |  |
|  |  | Physical inactivity | **Sattelmair et al. 2011 - Circulation** | **1.10 (0.96–1.30)** | **1.25 (1.09–1.45)** |
|  |  |  | Li et al. 2012 - Int J Environ Res Public Health | 1.18 (1.08–1.30) | 1.28 (1.18–1.39) |
|  |  |  | Sofi et al. 2008 - Eur Soc on Cardiol | 1.08 (1.01–1.14) | 1.11 (1.02–1.20) |
|  |  |  | Katzmarzyk et al. 2004 - Can J Appl Physiol | 1.45 (1.38–1.54) | 1.45 (1.38–1.54) |
|  |  |  |  |  |  |
|  |  | Smoker | **Mainous et al. 2006 - Am J Cardiol** | **1.6 (1.26–2.02)** | **3.22 (2.47–4.22)** |
|  |  | Occasional smoker | ***No data*** |  |  |
|  |  | Former smoker | **Mainous et al. 2006 - Am J Cardiol** | **0.99 (0.69–1.42)** | **1.15 (0.92–1.44)** |
|  |  |  |  |  |  |
|  | **Hypertension** | Obesity | **Guh et al. 2009 - BMC Public Health** | **1.84 (1.51–2.24)** | **2.42 (1.59–3.67)** |
|  |  | Overweight | **Guh et al. 2009 - BMC Public Health** | **1.28 (1.10–1.50)** | **1.65 (1.24–2.19)** |
|  |  |  |  |  |  |
|  |  | Physical inactivity | **Warburton et al. 2010 - Int J Behav Nutr Phys Act** | **1.47 (1.11–2.70)** | **1.47 (1.11–2.70)** |
|  |  |  | Katzmarzyk et al. 2004 - Can J Appl Physiol | 1.30 (1.16–1.46) | 1.30 (1.16–1.46) |
|  |  | Insufficient consumption of fruits and vegetables | No association |  |  |
|  |  |  |  |  |  |
|  |  | Smoker | **Harlperin et al. 2008 - Am J Hypert** | 1.15 (1.03, 1.27) | 1.15 (1.03, 1.27) |
|  |  | Former smoker | **Harlperin et al. 2008 - Am J Hypert** | 1.08 (1.01, 1.15) | 1.08 (1.01, 1.15) |
|  | |  |  |  |  |
| ***4) Metabolic diseases*** | |  |  |  |  |
| **Type 2 diabetes** | | Obesity | **Abdullah et al, 2010 - Diabetes Res Clin Pract** | **6.48 (5.17–8.13)** | **8.38 (5.46–12.85)** |
|  |  |  | Guh et al. 2009 - BMC Public Health | 6.74 (5.55-8.19) | 12.41 (9.03–17.06) |
|  |  |  |  |  |  |
|  |  | Overweight | **Abdullah et al. 2010 - Diabetes Res Clin Pract** | **2.63 (2.09–3.32)** | **3.69 (2.52–5.40)** |
|  |  |  | Guh et al. 2009 - BMC Public Health | 2.40 (2.12–2.72) | 3.92 (3.10–4.97) |
|  |  |  |  |  |  |
|  |  | Physical inactivity | **Jeon et al. 2007 - Diabetes Care** | **1.20 (1.11–1.32)** | **1.20 (1.11–1.32)** |
|  |  |  | Katzmarzyk et al. 2004 - Can J Appl Physiol | 1.50 (1.37–1.63) | 1.50 (1.37–1.63) |
|  |  |  |  |  |  |
|  |  | Insufficient consumption of fruits and vegetables | **Hamer et al. 2007 – J Hypertens** | **1.04 (0.85–1.27)*** | **1.04 (0.85–1.27)*** |
|  | |  |  |  |  |
| ***5) Musculoskeletal diseases*** | |  |  |  |  |
|  | **Osteoarthritis (Arthrosis)** | Obesity | **Guh et al. 2009 - BMC Public Health** | **4.20 (2.75–6.41)** | **1.96 (1.88–2.04)*** |
|  |  | Overweight | **Guh et al. 2009 - BMC Public Health** | **2.76 (2.05–3.70)** | **1.80 (1.75–1.85)*** |
|  |  |  |  |  |  |
|  | ***Osteoporosis*** | Physical inactivity ( | **Katzmarzyk 2004 - Can J Appl Physio** | **1.59 (1.40–1.80)** | **1.59 (1.40–1.80)** |
|  |  |  |  |  |  |

**Table S2. Activities targeted by the healthy lifestyle habits promotion strategy and their sources of funding, Quebec 2010-2011. Canada 2016**

| **Activity category** | **Activity subcategory and funding source** | **Examples of interventions** | **Costs (2010–2011 $)** |
| --- | --- | --- | --- |
| 1- Activities related to the implementation and coordination of the PAG (Plan d'action gouvernemental) for healthy habits promotion and prevention of weight-related problems | **Standardized costs of the PNSP (Plan National de Santé Publique)**: Human resources needed to implement the prevention and promotion activities funded by the PNSP budget | Implementation, coordination and support activities related to healthy habits promotion, food security, and smoking cessation | **$33.02M** in standardized costs for PAG activities^1^  $32.32M after excluding costs of counseling related to fluoride use and adding in food security activities |
| 2- Activities funded by Québec en forme (QeF) | Funding from the Quebec government and the Chagnon Foundation (50%) for projects dealing with healthy lifestyle habits in children age 17 and under (no funding for the public health network) | **Programs:** À pied, à vélo, ville active [Walking and cycling, a city on the move]; Réseau du sport étudiant du Québec (RSEQ) [Quebec student sport network]; Bien dans sa tête, bien dans sa peau [Healthy mind, healthy body].  **Partners**: RSEQ, Sports-Québec, Le Grand défi Pierre Lavoie | A total of  **$40M** annually for 10 years  In 2010-2011: **$39,741M^2^**   - $27.22M: Support to local communities and regional projects - $9.92M: Support to provincial projects - $4.60M: Administrative and general costs |
| 3- Other prevention activities and programs funded by the government of Quebec to help achieve the objectives of the PNSP and PAG | **Ministry of Health and Social Services (MSSS)**  a) Anti-smoking activities  b) Publicity campaigns | a) Anti-smoking activities (not including medical acts or the reimbursement of smoking cessation aids) | **a) Anti-smoking: $17M in 2011–2012^3^** *We assume costs were similar in 2010/11*  **b) Publicity campaigns**: numbers not available |
|  | **Ministry of Education, Leisure and Sports (MELS)** | **Kino-Québec prevention activities:** Funded by the MELS and the MSSS | **$1.36 M given to the regional health agencies^4^** |
|  | **Quebec Ministry of Transport (MTQ)** | Subsidies to promote the use of alternative means of transportation | **$11M** invested in the government program for support to non-automobile transportation alternatives^5^ |
| 4- Activities funded by the federal level |  | Programs: La gang allumée, Visez santé, Diabète Québec, Équilibre | **- Diabetes**: $418,000 from Public Health Agency of Canada (PHAC) for community projects^6^  **- Other unknown investments:** minimal compared to other sources |
| 5- Activities or programs funded by other organizations or foundations | RSEQ: funded by the MELS and several other Canadian and Quebec partners | Programs: De facto; Iso-Actif; Bien dans sa tête, bien dans sa peau | **$6.59M in 2010–2011^7^** |
| Total Costs |  |  | **≈ $110M** |

References:

^1^DGSP, 2010; ^2^QeF 2011; ^3^MSSS,2012; ^4^DGSP, 2011; ^5^MTQ, 2008; ^6^PHAC,2011 ^7^RSEQ, 2011.

PAG: Plan d’action gouvernemental [Government Action Plan]; PNSP: Programme nationale de santé publique [Quebec Public Health Program

**Table S3. Impact of discounting effects and accounting for increase in healthcare expenditures on risk factor costs and break-even point. Canada 2016**

|  | Delay between the effects occurs ($ attributable to the disease) | | | | |
| --- | --- | --- | --- | --- | --- |
|  | No discounting | 5 years | 10 years | 15 years | 20 years |
| Discounting effects (savings from disease avoidance) at 3.5% | $1,958,216,550 | $1,648,765,790 | $1,388,216,554 | $1,168,841,088 | $984,132,832 |
| Discounting and accounting for increase in health expenditures ($) | $1,958,216,550 | $2,044,850,025 | $2,135,316,252 | $2,229,784,796 | $2,328,432,723 |
| Break-even point (%) | 5.62% | 5.38% | 5.15% | 4.93% | 4.72% |

**Sensitivity analyses methodology**

To assess the robustness of the results and the associated uncertainty, we calculated an average RR estimate and its confidence intervals (95%) with all selected RRs through the literature review (see above) using bootstrapping methods (1,000 samples) for each of the associations between exposure and the selected chronic diseases. We then calculated best and worst case threshold estimates. First, in our model, we included the lower bound of estimate for each association between exposure and the selected chronic diseases, in combination with the highest possible cost of the HLHP strategy. Second, we included the higher bound of the probabilistic RR estimate for each association between exposure and the selected chronic diseases, in combination with the lowest possible cost of the HLHP strategy. Finally, we performed sensitivity analyses to assess the impact on the estimated effectiveness level (break-even point) of using different discount rates, such as 1.5% (recommended by NICE (2012) for health effects) and 5% (recommended by CADTH - Canadian Agency for Drugs and Technologies in Health - (2006) in Canada).

**Sensitivity analyses results**

We performed sensitivity analyses to assess the results’ sensitivity to variability in: 1) RR estimates, 2) total costs of the HLHP strategy, and 3) the discount rate used to assess future healthcare costs.

As presented in Table S5, we assessed a probabilistic RR estimate and 95% confidence intervals for each association between risk factors and the selected diseases. Sensitivity analysis using probabilistic RRs (bootstrapping) produced a similar base case estimate (5.60%) and a confidence interval of 4.14% to 9.35%. Our best and worst case analyses showed variation in the break-even point ranging from 3.38% (lower HLHP strategy cost estimate and higher costs attributed to risk factors) to 12.75% (higher HLHP strategy cost estimate and lower costs attributed to risk factors), indicating significant levels of uncertainty regarding HLHP strategy costs and the RR estimate parameters (Table S4).

Finally, Table S6 presents results using different discount rates to assess future costs of diseases showed the threshold to be moderately sensitive to the use of different discount rates, thus indicating that discounting at the rate proposed by CADTH (5%) would have a limited impact on the threshold.

**Table S4. Sensitivity analyses – Impact of variations in RR estimates and in costs of the HLHP strategy on the break-even point. Canada 2016**

|  | **Variations in HLHP strategy costs** | | |
| --- | --- | --- | --- |
|  | $90,000,000 | $110,000,000 | $150,000,000 |
| RR LCI | 7.65% | 9.35% | 12.75% |
| RR estimate | 4.58% | 5.60% | 7.63% |
| RR UCI | 3.38% | 4.14% | 5.64% |

**Table S5: Bootstrap relative risk estimates used for sensitivity analyses. Canada 2016**

| **Disease** | **Risk factor** | **Bootstrap estimates*** | | |
| --- | --- | --- | --- | --- |
|  |  | RR | LCI | UCI |
| **COPD** | Smoker | 2.435 | 1.639521 | 3.230479 |
|  | Occasional smoker | 1.58 | 1.064798 | 2.095202 |
|  | *Former smoker* | 2.01 | 1.3104 | 2.7096 |
|  | Insufficient consumption of fruits and vegetables | 1.895 | 1.091357 | 2.698643 |
| **Asthma** | Smoker | 1.50 | 1.222708 | 1.777292 |
|  | Occasional smoker | NA |  |  |
|  | Obesity | 1.430 | 1.209969 | 1.650031 |
|  |  |  |  |  |
| **Trachial, broncheal and lung cancers** | Smoker | 9.53 | 8.745358 | 10.31464 |
|  | *Former smoker* | NA |  |  |
|  | Insufficient consumption of fruits and vegetables | NA |  |  |
|  |  |  |  |  |
| **Breast cancer** | Physical inactivity | 1.245 | 1.007377 | 1.482623 |
|  | Insufficient consumption of fruits and vegetables | NA |  |  |
| ***Postmenopausal breast cancer*** | Obesity | 1.141 | 1.126278 | 1.153722 |
|  | Overweight | NA |  |  |
| **Cancer colorectal** | Obesity | 1.65 | 1.231169 | 2.068831 |
|  | Overweight | NA |  |  |
|  | Physical inactivity | 1.355 | 1.062897 | 1.647103 |
|  |  |  |  |  |
| **Stroke** | Obesity | 1.385 | 1.166351 | 1.603649 |
|  | Overweight | 1.14 | 1.013476 | 1.266524 |
|  | Insufficient consumption of fruits and vegetables | NA |  |  |
|  | Physical inactivity | 1.25 | 1.085556 | 1.414444 |
|  | Smoker | NA |  |  |
|  | Former smoker | NA |  |  |
| **Ischemic heart disease** | Obesity | NA |  |  |
|  | Overweight | NA |  |  |
|  | Insufficient consumption of fruits and vegetables | NA |  |  |
|  | Physical inactivity | 1.19 | 1.011288 | 1.268712 |
|  | Smoker | NA |  |  |
|  | *Former smoker* | NA |  |  |
| **Hypertension** | Obesity | NA |  |  |
|  | Overweight | NA |  |  |
|  | Physical inactivity | NA |  |  |
|  | Insufficient consumption of fruits and vegetables | NA |  |  |
|  | Smoker | NA |  |  |
|  | Former smoker | NA |  |  |
| **Type 2 diabetes** | Obesity | 6.61 | 6.422003 | 6.797997 |
|  | Overweight | 2.515 | 2.357391 | 2.67261 |
|  | Physical inactivity | 1.354 | 1.150126 | 1.549874 |
|  | Insufficient consumption of fruits and vegetables | NA |  |  |
| **Osteoarthritis (arthroses)** | Obesity | NA |  |  |
|  | Overweight | NA |  |  |
| ***Osteoporosis*** | Physical inactivity | NA |  |  |

* When these probabilistic RR estimates were used in our model, the break-even point was 5.60% (95% confidence interval of 4.14% to 9.35%).

**Table S6: Impact of variation in RR estimates and in costs of the HLHP strategy on the break-even point. Canada 2016**

|  | **Time to occurrence of effects (break-even threshold %)** | | | | |
| --- | --- | --- | --- | --- | --- |
|  | No discounting | 5 years | 10 years | 15 years | 20 years |
| Threshold if discount rate = 1.5 (%) | 5.60% | 4.86% | 4.22% | 3.67% | 3.19% |
| Threshold if discount rate = 3.5 (%) | 5.60% | 5.36% | 5.13% | 4.92% | 4.71% |
| Threshold if discount rate = 5 (%) | 5.60% | 5.76% | 5.93% | 6.10% | 6.28% |

**References :**

1. Direction générale de la santé publique (DGSP). (2010). Coûts normés du Programme national de santé publique 2003-2012 – Mise à jour 2008. Version finale pour l’allocation des ressources 2010-2011 [Stardard costs for the National Public Health Program 2003-2012, updated 2008. 2010-2011 ressource allocation final version]. Québec: Ministère de la Santé et des Services.
2. Québec en forme (QeF). (2011). États financiers de Fonds Québec en Forme [Financial statements of the Québec en forme Foundation]. Sainte-Foy-Silleray-Cap-Rouge, QC: QEF. Available at: <http://www.quebecenforme.org/media/72075/etats_financiers_31032011.pdf>
3. Ministère de la santé du Québec, 2012. Data not published.
4. Direction générale de la santé publique (DGSP), 2011. Attribution budgétaire pour le programme Kino-Québec [Budgetary attribution for Kino-Quebec Program]. Not published.
5. Ministère des Transports du Québec (MTQ). (2008). Programmes d’aide au transport collectif : Politique québécoise du transport collectif [Public transit assistance program: Quebec public transit policy]. Québec: MTQ. Available at: <http://www.mtq.gouv.qc.ca/portal/page/portal/Librairie/Publications/fr/ministere/programmes_aide/prog_aide_tc_pol_queb_transp_coll.pdf>
6. Public Health Agency of Canada (PHAC). (2011). Diabetes community-based projects in Quebec. Ottawa: PHAC. Available at: <http://www.phac-aspc.gc.ca/media/nr-rp/2011/2011_1110-qc-fs-fi-eng.php>
7. Réseau du sport étudiant du Québec (RSEQ). 2011. Rapport annuel 2010-2011 [2010-2011 Annual Report]. Montreal: RSEQ. Available at: <http://www.rseq.ca/media/350825/rapport_annuel_2010-2011.pdf>
